# HIV RNA measurement in dried blood spots of HIV-infected patients in Thailand using Abbott m2000 system

**DOI:** 10.1371/journal.pone.0227929

**Published:** 2020-01-24

**Authors:** Woottichai Khamduang, Ampika Kaewbundit, Amonrat Duangmano, Sayamon Hongjaisee, Virat Klinbuayaem, Guttiga Halue, Apichat Chutanunta, Wasna Sirirungsi, Gonzague Jourdain, Nicole Ngo-Giang-Huong

**Affiliations:** 1 Department of Medical Technology, Faculty of Associated Medical Sciences, Chiang Mai University, Chiangmai, Thailand; 2 Infectious Diseases Research Unit, Division of Clinical Microbiology, Department of Medical Technology, Faculty of Associated Medical Sciences, Chiang Mai University, Chiangmai, Thailand; 3 Institut de Recherche pour le Développement (IRD), Chiangmai, Thailand; 4 Research Institute for Health Sciences, Chiang Mai University, Chiangmai, Thailand; 5 Sanpatong hospital, Chiangmai, Thailand; 6 Phayao hospital, Phayao, Thailand; 7 Samut Sakorn hospital, Samut Sakorn, Thailand; 8 Department of Immunology and Infectious Diseases, Harvard T.H. Chan School of Public Health, Boston, MA, United States of America; University of North Carolina at Chapel Hill, UNITED STATES

## Abstract

World Health Organization recommends using dried blood spots (DBS) for HIV RNA viral load (VL) measurement whenever plasma processing is not convenient or feasible. DBS collected from 80 treatment-naïve HIV-infected patients presenting in three hospitals of two different regions of Thailand were shipped to a central laboratory along with corresponding plasma specimens. Viral load was measured in both DBS and plasma using the Abbott m2000 system. HIV RNA levels were strongly correlated (*r* = 0.94) with a mean of differences of 0.23 log_10_ copies/mL. Using the 1,000 copies/mL cut-off, the sensitivity of DBS was 97% (95%CI, 91–100%) and specificity was 75% (95%CI, 19–99%). DBS are useful to scale-up HIV RNA VL testing in settings with limited access to VL testing.

## Introduction

In 2014, the UNAIDS set the 90-90-90 targets to reach by 2020 to end the AIDS epidemic [1]. In Thailand, it is estimated that about 450,000 people were living with HIV, over 270,000 individuals were receiving antiretroviral therapy (ART) in 2015 [2] and 211,000 (78% of those on ART) benefited HIV RNA viral load (VL) measurement [3]. Monitoring plasma HIV RNA VL is key to assess the response to ART. However, due to the limited access to plasma HIV RNA measurements in some settings, WHO has recommended using dried blood spots (DBS) as an alternative to plasma specimens [4]. Indeed, DBS present several advantages which make their use particularly attractive whenever access to VL testing is difficult: it is simple to prepare and convenient for blood collection and transportation (shipping by mail to a central testing laboratory) with low cost, and small volume of blood is needed. In addition, fingerpick blood specimen can be applied onto the DBS [5].

Previous studies have shown that DBS can be used to reliably measure HIV RNA VL with different platforms [6–9]. Routine monitoring of HIV RNA VL using DBS has been successfully implemented in resource-limited settings such as Angola, Tanzania, and Cameroon [10–12]. Several studies have evaluated the performances of the Abbott m2000 platform for the measurement of RNA in DBS versus plasma [5, 13–16]. Those studies were conducted among different populations (naïve or on ART) and used different DBS processing protocols. The sensitivity of DBS at the 1,000 cp/mL cut-off ranged between 76–100%, the specificity between 90–96% and the mean differences between Plasma-DBS were between 0.02–1.1 log_10_ cp/mL. These results indicate that the Abbott m2000 can perform well on DBS in different settings. In Thailand, HIV RNA are usually measured in plasma specimens and tests are mostly performed in centralized laboratories using commercial assays. In order to increase access to HIV RNA measurements for people living in remote areas, HIV RNA VL measurement in DBS was compared to measurement in plasma of treatment-naïve HIV-infected patients presenting at hospitals in two different regions of Thailand.

## Materials and methods

### Study setting and participants

Consenting HIV-infected individuals at least 18 years old, antiretroviral-naïve or having history of prevention of HIV mother-to-child transmission were enrolled at Sanpatong hospital (Chiangmai province) or Phayao hospital (Phayao province) in Northern region or Samut Sakorn hospital (Samut Sakorn province) in Central region of Thailand. Written informed consents were obtained from all participants for blood testing. Ethical approval was obtained from the Ethic committee of the Faculty of Associated Medical Sciences, Chiang Mai University (Reference no. AMSEC-58EX-038). Local ethical approval was also obtained from each hospital (The Ethic committees of Sanpatong hospital, of Phayao hospital, and of Samut Sakorn hospital).

### Preparation of DBS and plasma

Six milliliters of whole blood were collected by venous puncture and transferred into an EDTA tube. Dried blood spots were prepared as previously described [17, 18]. Briefly, whole blood was dispensed in 5 spots of 50 μL each onto a Whatman 903® Protein Saver Card (Whatman, UK) and dried overnight at ambient temperature (25–30°C). Once dried, filter papers were stored in plastic zip-lock dispensing bags with a silica desiccant and kept at -20°C until shipment. The remaining EDTA whole blood specimens were centrifuged at 1,500 g for 15 minutes and plasma immediately transferred to sterile tubes and stored at -20°C until shipment. Both specimens were shipped with dry ice to the reference laboratory IRD UMI174-PHPT, Faculty of Associated Medical Sciences, Chiang Mai University, Chiangmai. All specimens were then stored at -80°C until testing.

### HIV viral load measurement in DBS specimens

Blood was eluted from 2 spots of dried blood (equivalent to 100 μL of whole blood) placed in 2 mL of lysis buffer for 3 hours with gentle rotation. The eluted blood was then transferred to a new tube and centrifuged at 2,000 rpm (385 x g) for 10 minutes to remove the pieces of filter papers. The supernatant was further processed for automated isolation of nucleic acid using the Abbott m2000sp (Abbott Molecular Inc., USA) and the protocol “1 mL DBS HIV-1 RNA” according to the manufacturer’s instructions. Quantification was performed using the Abbott molecular m2000rt system (Abbott Molecular Inc., USA) with the detection threshold of 550 cp/mL. No manual data correction was required as there was an automatically software-embedded correction factor which took into account the input volumes used. HIV RNA VL obtained from DBS specimens was reported as copies per milliliter of plasma (cp/mL).

### HIV viral load testing from plasma specimens

Plasma HIV RNA VL testing was performed using the Abbott m2000sp and Abbott molecular m2000rt system (Abbott Molecular Inc., USA) and the “plasma HIV-1 RNA protocol” according to the manufacturer’s instructions. The lower limit of detection (LOD) in plasma is 40 cp/mL. HIV RNA VL obtained from plasma specimens was reported as cp/mL.

Plasma HIV RNA VL testing was performed using the Abbott m2000sp and Abbott molecular m2000rt system (Abbott Molecular Inc., USA) and the “plasma HIV-1 RNA protocol” according to the manufacturer’s instructions.

### Statistical analysis

The results of HIV RNA VL in DBS and plasma specimens are expressed as median and interquartile range (IQR). Undetectable HIV RNA VL results were considered as 1.30 log_10_ cp/mL (half of the limit of detection, LOD: 1.60 log_10_ cp/mL), detectable HIV RNA VL but lower than the LOD as 1.60 log_10_ cp/mL. Correlation between DBS HIV RNA VL and plasma VL was analyzed using Pearson’s correlation coefficient. The degree of agreement (concordance) between DBS HIV RNA VL and plasma HIV RNA VL results was assessed with Bland-Altman analysis. Specificity, sensitivity, and positive/negative predictive values of DBS HIV RNA VL testing versus plasma, as reference, were evaluated. All analyzes were performed using STATA^™^ version 14.1 software (Statacorp, College Station, TX). Statistical significance was considered when P-value was <0.05.

## Results

### Patient characteristics

Eighty patients were enrolled in this study. Sixty (75%) were male and 20 (25%) were female. The median age was 33.7 years old with an interquartile range (IQR) of 24.1–42.7. Seventy-six (95%) patients were antiretroviral-naïve and 4 (5%) patients had previously received antiretroviral drugs for prevention of mother-to-child transmission of HIV (Table 1).

**Table 1:** Baseline characteristics of study population.

Baseline characteristics (N = 80)		Values (% or IQR)
Gender	- Male	60 (75%)
	- Female	20 (25%)
DBS collected from	- Phayao hospital	22 (27.5%)
	- Samut Sakorn hospital	20 (25%)
	- Sanpatong hospital	38 (47.5%)
Age, years old	Median (IQR)	33.7 (24.1–42.7)
Antiretroviral drug received	- No	76 (95%)
	- Yes*	4 (5%)
Plasma HIV VL (log_10_ cp/mL)**	<1.60	1 (1.3%)
	1.60–1.99	0 (0%)
	2.00–2.99	3 (3.8%)
	3.00–3.99	5 (6.3%)
	4.00–4.99	30 (37.5%)
	5.00–5.99	35 (43.8%)
	6.00–6.99	6 (7.5%)
	Median (IQR)	5.05 (4.43–5.61)

*They had received antiretroviral drugs according to prevention of mother-to-child transmission of HIV

**Measured by the Abbott molecular m2000rt system

### HIV-1 RNA measurement in plasma and DBS using Abbott molecular m2000 system

Median of plasma HIV RNA VL was 5.05 (IQR: 4.43–5.61) log_10_ copies/mL. Median HIV RNA VL in DBS was 4.80 (IQR: 4.13–5.39) log_10_ cp/mL. One patient (1.3%) had undetectable HIV RNA in both DBS and plasma specimens. Two (2.5%) patients had detectable HIV RNA VL (2.18 and 2.19 log_10_ cp/mL) in plasma specimens but undetectable in DBS. (Refer to the Supplementary data S1 Table)

### Correlation and concordance between DBS and plasma viral load results

**Fig 1:**
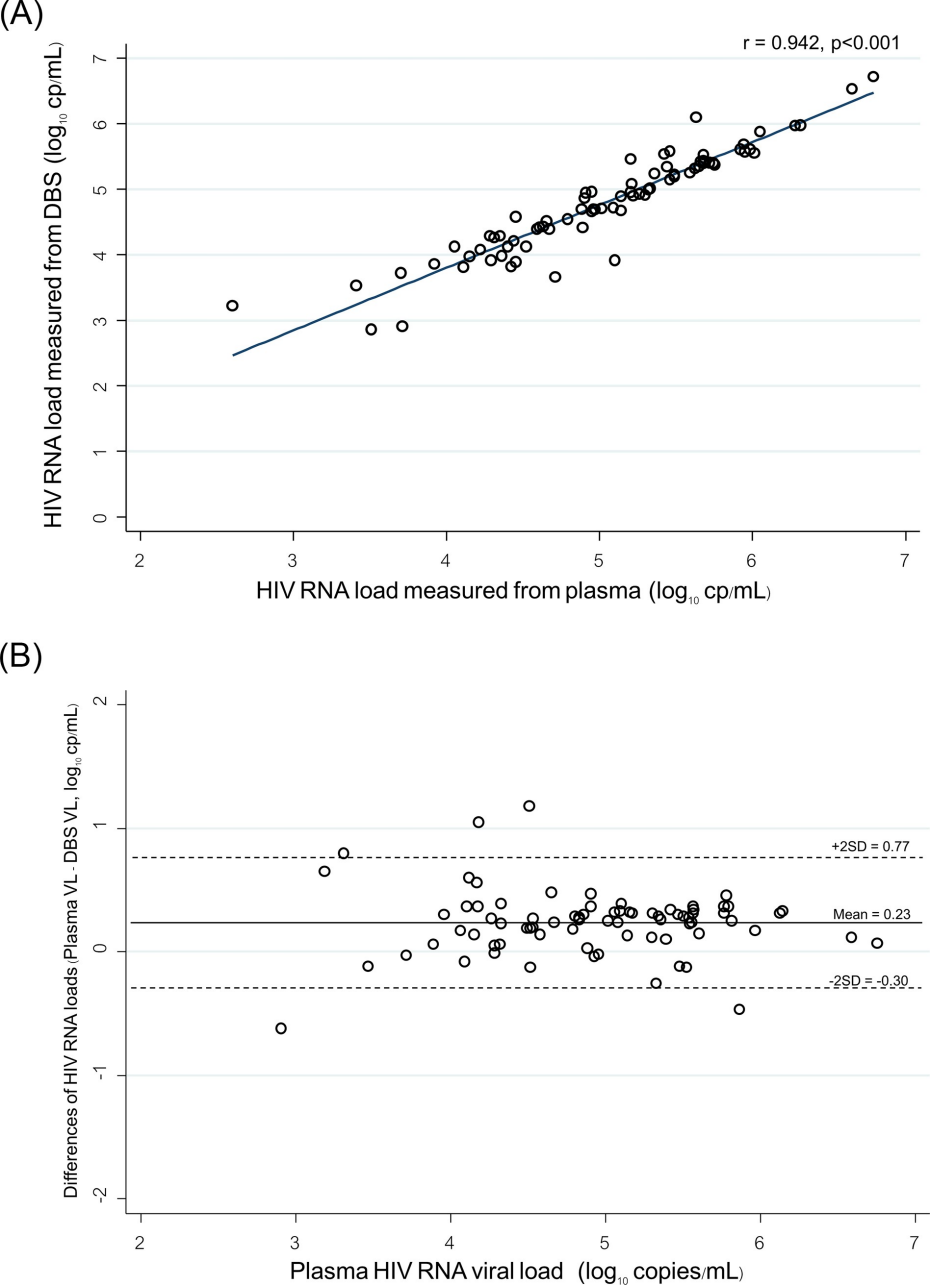
(A) Regression analysis of HIV RNA levels in paired plasma and DBS specimens from 80 participants. (B) Bland-Altman analysis of agreement between HIV RNA levels in 80 paired plasma and DBS specimen. The horizontal lines represent the mean difference and +2 standard deviations (SD).

HIV RNA levels in plasma and in DBS were strongly correlated (correlation coefficient (*r*) of 0.94 and coefficient of determination (*r*^*2*^) of 0.89 (Fig 1A), excluding the values below the lower limit of quantitation. The concordance between plasma HIV RNA and DBS HIV RNA results is shown in the Bland-Altman plot (Fig 1B). The mean HIV RNA level was slightly higher in plasma than in DBS with the mean of differences of 0.23 log_10_ copies/mL and 2 standard deviation (2SD) of 0.53. Sixty (78%) specimens had a difference of plasma and DBS HIV RNA VL within a range of ±0.25 log_10_ copies/mL, and 72 (94%) and 77 (100%) were within a range of ±0.5 and of ±1.0 log_10_ copies/mL, respectively.

Among the 4 patients with plasma HIV RNA VL <1,000 copies/mL, 3 patients had also HIV RNA VL <1,000 copies/mL in DBS and 1 patient had HIV RNA VL ≥1,000 cp/mL in DBS (3.22 log_10_ cp/mL in DBS, 2.60 log_10_ in plasma). Of 76 patients with plasma HIV RNA VL ≥1,000 copies/mL, 74 had also HIV RNA VL ≥1,000 copies/mL in DBS. Sensitivity of DBS HIV RNA measurement was 97% (95%CI, 91–100%) and specificity was 75% (95%CI, 19–99%) as compared to plasma HIV RNA measurement. Positive predictive value of DBS VL was 99% (95%CI, 93–100%) and negative predictive value was 60% (95%CI, 26–87%). The accuracy was 96% (95%CI, 89–99%).

## Discussion

In this study, HIV RNA VL results in DBS specimens were strongly correlated to VL results in plasma specimens, *r* = 0.94. HIV RNA levels measured in DBS were slightly lower than in plasma (0.23 log_10_ copies/mL). This could be due to the higher input volume of plasma (1mL) as compared to the DBS (0.1 mL of whole blood) [19] and possibly the higher rate of HIV RNA recovery in plasma than in DBS during the extraction. The higher level in plasma may also be due to the lack of hematocrit adjustment as reported in other studies [19, 20]. This slightly lower average HIV RNA levels in DBS than in plasma has also been observed in other studies with average differences ranging between 0.07–0.76 log_10_ copies/mL [5, 12, 16, 21, 22]. The smaller volume of blood tested in DBS might also explain that 2 DBS were undetectable while the corresponding plasma specimens had low HIV RNA VL (2.18 and 2.19 log_10_ cp/mL) which were below the threshold of detection in DBS.

We assessed the sensitivity of HIV RNA detection in DBS using the cut-off at 1,000 copies/mL of plasma, the usual cut-off to consider for treatment failure. We found similarly to studies conducted in other settings [5, 16, 23] a very high sensitivity of Abbott system to detect HIV RNA in DBS (97%) with a very high positive predictive value (99%). Also, the accuracy of HIV RNA measurement in DBS was very high (96%). All these results suggest that testing HIV RNA in DBS using the Abbott system will be useful to detect treatment failure in settings where plasma HIV RNA could not be easily performed. However, using DBS for monitoring the response to ART treatment have some drawbacks to consider: 1) false positive results due to the detection of cellular nucleic acids with the risk of diagnosing a virological failure in a patient who is actually controlling HIV replication or 2) false negative results due to the degradation of RNA in DBS stored in bad conditions with the risk of diagnosing viral suppression. In many resource-limited settings, the high humidity level in environment may impact on the preparation and processing of DBS. Accurate HIV RNA testing results require that DBS be completely dried before packing and shipped in plastic bag with desiccants.

Our study has some limitations. The correlation was done with DBS not shipped by regular post which may not reflect the real life. However, this study and other studies reported no or few losses of HIV RNA after shipping at various conditions, especially in whole blood specimens with high HIV RNA VL [24, 25]. Another limitation was that most of the specimens in this study were obtained from treatment-naïve patients who had a very high HIV RNA VL. Due to the low number of specimens (n = 4) with viral load below 1,000 copies/mL in plasma, the specificity was 75% with a wide confidence interval. In other studies conducted on high numbers of DBS with low HIV VL, specificity of Abbott system ranged between 95–96% [5, 16, 23].

In conclusion, our results suggest that DBS have a very good sensitivity at the threshold of 1,000 copies/mL and can be used for HIV RNA VL measurement in Thailand. Using DBS with Abbott system will increase the access to HIV RNA VL testing for HIV-infected patients who cannot benefit regular routine treatment monitoring. Scaling-up of HIV RNA VL will contribute to achieve the UNAIDS 90-90-90 targets to reach to end the AIDS epidemic.

## Supporting information

S1 Table All individual essential values used in the manuscript. (PDF)
