# Supplementary material for: HIV RNA measurement in dried blood spots of HIV-infected patients in Thailand using Abbott m2000 system
Source: PLoS One. 2020 Jan 24;15(1):e0227929. doi: 10.1371/journal.pone.0227929 (PMC6980635; doi:10.1371/journal.pone.0227929)
Supplement: S1 Table — (PDF) [file pone.0227929.s001.pdf]

Site	Identification	Gender	Age	ART-experiented	ART type	Regimen	Plasma HIV VL log	DBS HIV VL log
05	VLD001	Male	18	No			6.01	5.55
05	VLD002	Male	24.1	No			5.74	5.40
05	VLD003	Male	37.5	No			5.44	5.34
05	VLD004	Female	47.6	No			5.68	5.44
05	VLD005	Female	33.4	No			4.22	4.08
05	VLD006	Male	52	No			5.20	4.96
05	VLD007	Male	22.3	No			3.51	2.86
05	VLD008	Male	39.5	No			5.09	4.72
05	VLD009	Male	28.1	No			6.05	5.88
05	VLD010	Male	44.6	No			5.22	4.90
05	VLD011	Male	23.1	No			5.46	5.15
05	VLD012	Male	36.8	No			5.49	5.20
05	VLD013	Male	24.5	No			5.30	4.91
05	VLD014	Male	48.1	No			3.71	2.91
05	VLD015	Female	32.8	Yes	PMTCT	AZT	5.42	5.54
05	VLD016	Male	22	No			4.44	4.21
05	VLD017	Male	24.8	No			4.95	4.97
05	VLD018	Male	21.2	No			4.59	4.40
05	VLD019	Male	29.9	No			5.68	5.53
05	VLD020	Male	62.8	No			2.18	1.30
05	VLD021	Male	50.1	No			5.14	4.67
05	VLD022	Male	33	No			5.32	5.00
35	VLD031	Male	35.2	No			6.28	5.97
35	VLD032	Male	57.5	No			5.72	5.41
35	VLD033	Male	51.3	No			4.89	4.41
35	VLD034	Female	40.1	Yes	PMTCT	not available	4.61	4.42
35	VLD035	Female	42.2	No			4.29	3.92
35	VLD042	Male	47.7	No			4.91	4.95
35	VLD043	Male	45.5	No			5.98	5.61
35	VLD044	Female	32.8	No			5.14	4.89
35	VLD045	Male	32.1	No			5.94	5.69
35	VLD046	Female	43.2	No			5.59	5.25
35	VLD047	Male	28.1	No			4.52	4.13
35	VLD048	Male	21.6	No			5.33	5.02
35	VLD049	Female	35.5	No			4.96	4.70
35	VLD050	Male	34.7	No			3.92	3.86
47	VLD051	Male	22	No			4.42	3.82
47	VLD052	Male	29	No			3.41	3.53
47	VLD053	Male	40.4	No			5.49	5.23
47	VLD054	Male	18.1	No			5.75	5.38
47	VLD055	Male	20.4	No			4.67	4.40
47	VLD056	Male	34.6	No			4.28	4.29
47	VLD057	Male	24.1	No			4.97	4.69
47	VLD058	Male	21.1	No			4.65	4.51
47	VLD059	Male	19.5	No			1.30	1.30
47	VLD060	Male	18.2	No			4.45	3.89
47	VLD061	Female	28.1	Yes	PMTCT	COMBID + LPV/RT	2.98	1.30
47	VLD062	Male	22.4	Yes	PMTCT	COMBID + LPV/RT	4.45	4.58
47	VLD063	Female	35.4	No			5.63	6.10

47	VLD064	Female	37.3	No			5.20	5.46
47	VLD065	Male	34	No			4.05	4.13
47	VLD066	Male	50.3	No			4.71	3.66
47	VLD067	Female	43.3	No			3.70	3.73
47	VLD068	Male	52.8	No			6.79	6.72
47	VLD069	Male	52.5	No			4.11	3.81
47	VLD070	Male	23.5	No			5.10	3.92
47	VLD071	Female	19.5	No			4.79	4.55
47	VLD072	Male	22.6	No			4.90	4.87
47	VLD073	Male	36.6	No			5.68	5.40
47	VLD074	Female	34.6	No			5.36	5.24
47	VLD075	Female	46.6	No			6.65	6.53
47	VLD076	Male	18.6	No			4.35	4.29
47	VLD078	Male	24.8	No			4.40	4.13
47	VLD079	Female	31.6	No			4.31	4.26
47	VLD080	Male	54	No			5.66	5.43
47	VLD081	Female	69.7	No			4.36	3.99
47	VLD089	Male	35.7	No			4.95	4.66
47	VLD090	Male	26.7	No			4.15	3.98
47	VLD091	Male	50.3	No			5.92	5.61
47	VLD092	Male	18.1	No			4.88	4.70
47	VLD093	Male	22.8	No			5.21	5.08
47	VLD094	Male	31	No			5.01	4.71
47	VLD095	Male	25.4	No			5.26	4.93
47	VLD096	Male	29.6	No			5.46	5.59
35	VLD101	Female	34.2	No			2.60	3.22
35	VLD102	Male	43.3	No			5.65	5.36
35	VLD103	Male	24.6	No			4.63	4.43
35	VLD104	Male	35.9	No			6.31	5.98
35	VLD105	Female	40.8	No			5.95	5.58
35	VLD106	Female	36.7	No			5.62	5.32
